# The importance of psychological support for end-stage renal disease dialyzed patients

**DOI:** 10.1192/j.eurpsy.2021.1923

**Published:** 2021-08-13

**Authors:** M. Regaya, B. Amamou, C. Boukhris, L. Gaha

**Affiliations:** Department Of Psychiatry, University of Monastir, Faculty of Medicine of Monastir, LR05ES10, Fattouma Bourguiba Hospital, Monastir, Tunisia

**Keywords:** Psychology, counseling, Healthcare, end-stage renal disease

## Abstract

**Introduction:**

End-Stage Renal Disease (ESRD) patients have difficult and challenging lifestyle due to the burden of the disease that, leads to numerous psychological issues. Regardless, healthcare providers usually focus on the somatic side and don’t take care of the psychological dimension.

**Objectives:**

Determine the effect of psychological support on the wellbeing of patients with ESRD.

**Methods:**

A survey sheet including sociodemographic and clinical data were distributed to two different groups. Psychological counseling was given to one of the groups, and wasn’t given to the other one.

**Results:**

Patients not receiving psychological support had higher levels of anxious and depressive symptoms and suicidal ideations compared to the group receiving psychological support that had lower levels of anxious, depressive symptoms and suicidal ideation.

**Conclusions:**

ESRD patients are at risk of developing all sorts of psychological issues. Which underlines the importance of the psychological support associated to the appropriate somatic care.

**Disclosure:**

No significant relationships.

